# Right Shoulder Luxatio Erecta: Inferior Shoulder Fracture Dislocation

**DOI:** 10.7759/cureus.43710

**Published:** 2023-08-18

**Authors:** Mathew George, Andrew Dekker, Neil Ashwood

**Affiliations:** 1 Medical School, University of Leicester, Leicester, GBR; 2 Trauma and Orthopaedics, University Hospitals of Derby and Burton, Burton-on-Trent, GBR; 3 Trauma and Orthopaedics, University Hospitals of Derby and Burton, Derby, GBR

**Keywords:** rare shoulder dislocation, hand behind head position, inferior shoulder dislocation, luxatio erecta humeri, luxatio erecta

## Abstract

Here, we present the case of a 68-year-old female presenting with an inferior shoulder dislocation of the glenohumeral joint. Accounting for a minority of all shoulder dislocations, the rarity of this injury makes it a unique and interesting presentation in patients. Following successful reduction and neurovascular assessment, the patient recovered adequately without any complications typically associated with this type of injury. The aim of this report is to outline the mechanism of action of the injury and describe the typical patient presentation alongside discussing management techniques and associated complications.

## Introduction

Luxatio erecta is the inferior dislocation of the glenohumeral joint and accounts for fewer than 0.5% of all shoulder dislocations [1], with most shoulder dislocations being anterior. Classically, it presents with a hyperabducted extremity, with the elbow flexed and the forearm pronated, i.e., hand behind head position. First described in 1859 by Middendorf and Scharm [2], it is typically associated with soft tissue injuries to the rotator cuff and fractures of the greater tuberosity, clavicle, and coracoid [2]. There is also a high risk of neurovascular compromise from the injury and iatrogenically during reduction [3].

In this report, we describe an inferior dislocation with an avulsed greater tuberosity. This report aims to outline the mechanism of action of injury, describe the typical patient presentation, and discuss the management techniques and complications of this rare injury.

## Case presentation

**Figure 1:**
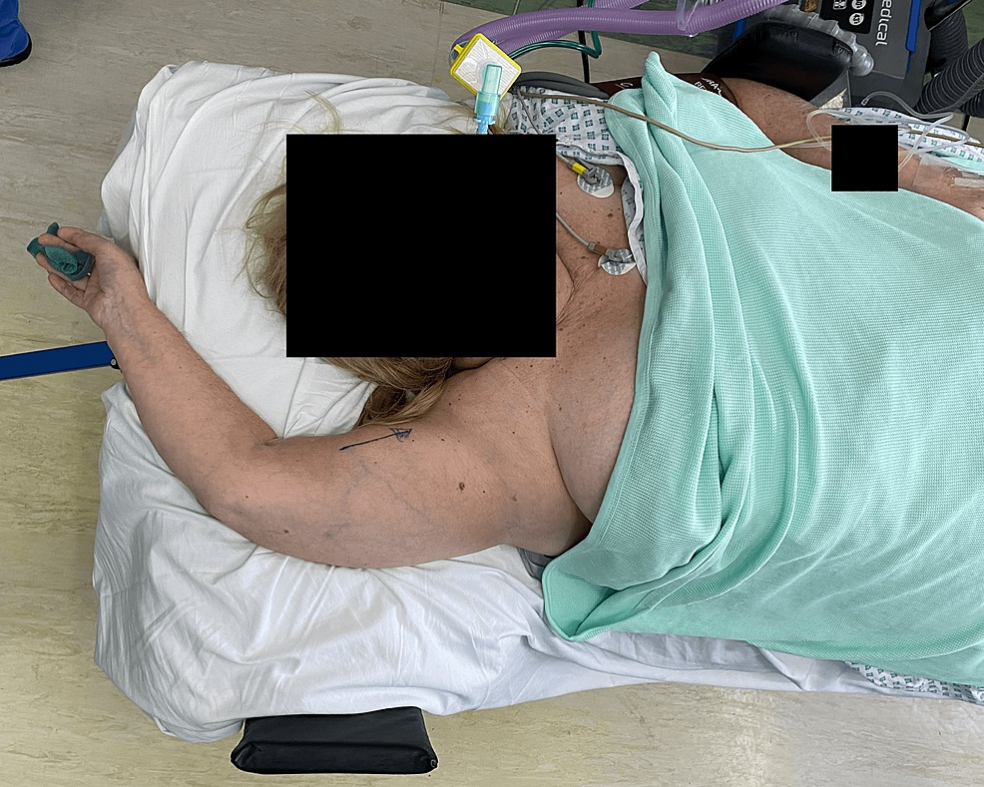
Typical presentation of a patient with the arm locked above the shoulder in hyperabduction, flexion of the elbow, and pronated forearm.

A 68-year-old female tripped over her grandson and tried to break her fall by falling on the end of a couch with a hyperflexed right arm. Since the fall, the patient was experiencing severe pain in the right shoulder and was unable to lower her right arm from a position above her head. On examination, her arm was locked overhead, with the elbow flexed and the forearm pronated in a typical luxatio erecta position (Figure 1).

**Figure 2:**
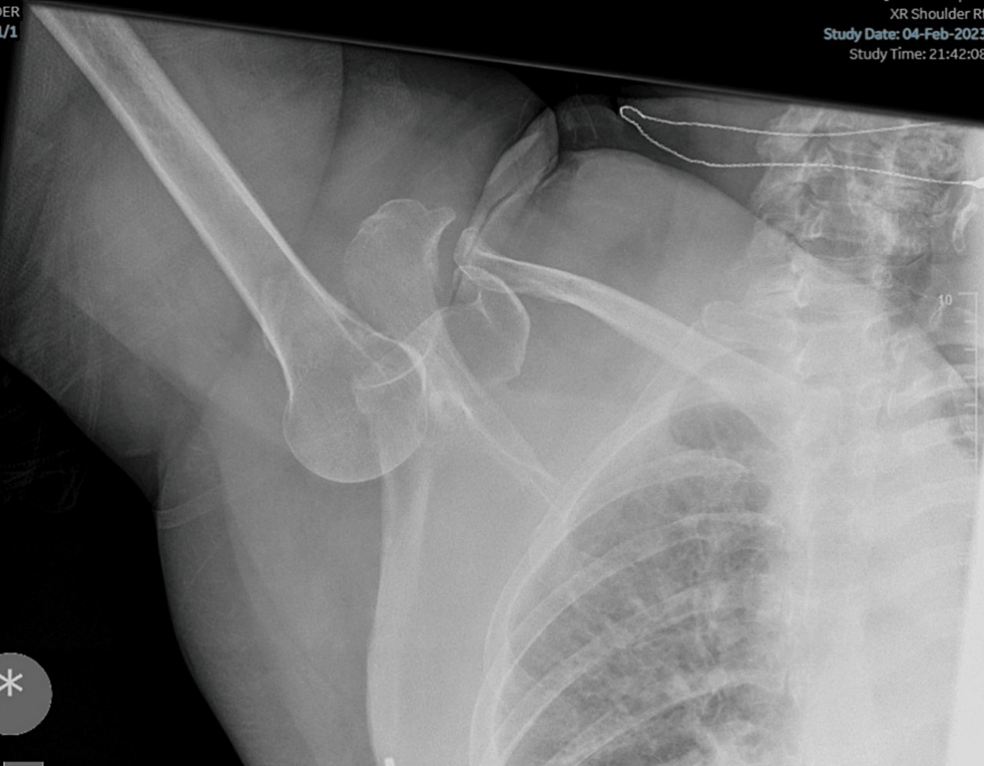
An anteroposterior X-ray showing the humeral head in an inferior subglenoid position with a greater tuberosity fracture.

There was no bruising, and her radial and ulna pulses were normal. Normal sensation and power to the median, radial, ulna, and axillary nerves were noted. There was no history of previous fractures. No head injuries were noted, and the patient was able to recall the events leading up to after the fall. X-rays revealed inferior fracture dislocation with avulsed greater tuberosity (large fragment) (Figure 2).

Management consisted of right shoulder closed manipulation under general anesthetics. The patient was laid supine on the table and gentle in-line traction with the arm above the head was applied, along with gentle external rotation and anteriorly directed pressure on the humeral shaft to disengage the humeral head anteriorly. Subsequently, further traction and manual pressure over the humeral head were applied with adduction, reducing the humeral head back into the glenoid fossa. No excessive force was required, and once the head of the humerus was reduced, the joint was stable through a full range of movement. A stable tuberosity position was achieved without requiring surgical fixation. A palpable radial pulse was identified, and the patient was placed in a sling. Following reduction, normal power and sensation to the median, radial, ulna, musculocutaneous, and axillary nerves were noted. The patient’s hand was warm and pink with a capillary refill time of under two seconds. It was explained to the patient that the procedure did not require open reduction or fixation and that she was fit for discharge with a sling to be reviewed in two weeks. On follow-up, X-rays showed no change to the tuberosity, and gentle mobilization supervised by a physiotherapist was advised with no restriction to the range of movement.-

## Discussion

There are two possible mechanisms of injury resulting in this rare inferior dislocation, as described by Davids and Talbot [4]. The most common mechanism is when the arm is in extension, hyperabduction, and external rotation (pronated), resulting in an indirect dislocation, as seen in our patient. The second is a direct force being applied to the shoulder from above, resulting in direct dislocation.

Closed reduction under anesthesia is the first line of management. Before and after reduction, it is important to check and document the neurovascular status due to the risk of neurovascular compromise. There are two reduction techniques that can be used to return the head of the humerus to its normal anatomical position. The more common one described by Freundlich [5] uses an opposite traction. The more recently described technique consists of a step maneuver whereby the luxatio erecta is forced into an anterior dislocation, and from there, the humeral head is reduced back into the glenoid fossa, as with a normal anterior dislocation [6]. As this is thought to reduce the risk of iatrogenic fracture, it was used in this case.

There is a high incidence of complications associated with luxatio erecta humeri dislocations. Fracture of the greater tuberosity or rotator cuff tear is seen in 80% of patients with this type of injury. Mallon et al. noted that 60% of patients reviewed in the literature had some degree of neurological compromise mostly associated with the axillary (circumflex) nerve [7]. However, these tend to resolve after successful reduction, but the time for resolution is variable. Only 3.3% of all noted cases lead to significant vascular compromise. Despite being low risk, this is still the highest noted for any type of shoulder dislocation. Other associated musculoskeletal injuries involve fractures to the inferior glenoid fossa, acromion, and clavicle. In our case, the patient had avulsion of the greater tuberosity which after closed reduction remained stable and did not require fixation.

## Conclusions

Luxatio erecta humeri represents the minority of all shoulder dislocations. Patients typically present with a characteristic position, with the arm hyperabducted, flexed at the elbow, and pronation of the forearm. It has a high incidence of complications, the major being fracture of the greater tuberosity, rotator cuff tears, and neurological compromise to the axillary nerve. First-line management is closed reduction under anesthesia, of which there are two commonly used techniques. Early reduction should be done to prevent complications. It is important to assess for neurovascular functions both before and after reduction, as a compromise to neurovascular structures can occur as a direct result of injury and because of reduction. Despite the potential for associated injuries, with prompt and successful reduction, the long-term prognosis is favorable, and patients are followed up through physio-mediated strength and conditioning.
